# Helping Institutionalised Children through a Trusting Relationship—Findings from a One-Year Psychosocial Intervention Programme

**DOI:** 10.3390/bs14070595

**Published:** 2024-07-13

**Authors:** Monika Misevičė, Lina Gervinskaitė-Paulaitienė, Sigita Lesinskienė

**Affiliations:** 1 Clinic of Psychiatry, Faculty of Medicine, Vilnius University, 01513 Vilnius, Lithuania; sigita.lesinskiene@mf.vu.lt; 2 Institute of Psychology, Faculty of Philosophy, Vilnius University, 01513 Vilnius, Lithuania; lina.gervinskaite@gmail.com

**Keywords:** attachment, disorganisation, institutionalised children, psychosocial interventions, Trust-Based Relational Intervention

## Abstract

Children growing up in institutions lack a stable relationship—essential for development. A significant proportion of them have disorganised attachment, which is associated with negative outcomes. Therefore, interventions aimed at improving attachment security are needed. We conducted a case series study (involving five participants aged 8–11 years) in a newly established psychosocial rehabilitation daycare centre to describe the changes in attachment security and mental health of institutionalised children after one year of attendance. The intervention consisted of individualised care focusing on staff building a trusting relationship with participants using the principles of Trust-Based Relational Intervention^®^ (TBRI). Measures such as the Child Attachment Interview, SDQ, CBCL6/18, TRF6/18, and clinical interviews were used to follow change. The descriptive data of five participants are presented. All five children improved in mental health, and for two participants, organisation of attachment changed. Three children experienced relational ruptures. The findings suggest that psychosocial interventions that focus on building trusting relationships may be beneficial in institutionalised children. For these children, given their life history, it can be damaging to experience repeated breakdowns. Thus, the practical implication is that any new relationship for them with an adult should be established after an assessment of whether the relationship can be sustained.

## 1. Introduction

“I will have parents now”, said a 9-year-old boy during the interview with the researcher. These words were perceived by the main researcher as expressing a fundamental need for attachment to a particular person. The aim of our research was to describe the impact of a psychosocial intervention on children in care, with a focus on how the intervention might affect children’s attachment security. During the study, two of our participants experienced life-changing events: one was forming a relationship with his future foster parents, and the other was preparing to return to his biological parents after spending most of his life in care. The remaining three participants experienced ruptured relationships with their temporary foster families. These were the real-life challenges that we faced in our study. In this paper, we present a study focusing on a descriptive case analysis of the data of five participants before entering a psychosocial rehabilitation programme and after one year of participation. We will describe in more detail the two cases where the organisation of attachment changed and discuss the practical implications of our findings.

### 1.1. The Impact of Institutional Care on the Attachment of Children and the Potential for Psychosocial Interventions to Promote Healing

Despite ongoing deinstitutionalisation efforts in Lithuania, we still have more than a thousand children growing up in institutions (according to SPIS (Information System for Social Family Support, Lithuania) data retrieved on 19 July 2023, 1310 children are in institutional care.). As authors, we adhere to the opinion that children should spend as little time as possible in institutions [1,2,3]. In accordance with the Convention on the Rights of the Child, the optimal environment for children is a safe family environment [4]. A body of research has consistently demonstrated the deleterious impact of institutional rearing on children’s physical health, cognitive abilities, social interactions, and mental well-being [2,5,6,7,8]. In this study, we focused on the impact of institutionalisation on each child’s attachment, as the psychosocial intervention studied also focused on improving attachment security. According to Johnson [6], “a child raised in an institution is typically deprived of the supportive, intensive, one-to-one relationship with a primary caregiver that is essential for optimal development”. This essential deprivation has consequences for the child’s attachment. Researchers have found 60 per cent disorganised attachment in institutionalised children, compared to 15 to 24 per cent in normative samples [3,9]. Vorria found [10] that children with institutional experience were still less secure than the comparison children at four years of age, and attachment insecurity was likely related to institutional deprivation. At the age of thirteen, the attachment of the same group was assessed using the Child’s Attachment Interview (CAI) method, and among the previously institutionalised adoptees, the rate of disorganisation dropped from 63 per cent in infancy to only 10 per cent in adolescence. The adoptee group also did not differ significantly from the comparison group in terms of attachment security [11]. Rutter et al. followed previously institutionalised children from Romania: a significant proportion of children developed the characteristics of disinhibited attachment, which persisted up to the age of 11 and was associated with an increased rate of dysfunction in other areas [12]. It is important to note that Rutter emphasises in his findings that the features of disinhibited attachment had only a weak association with attachment insecurity [12]. We can hypothesise that these children may be classified as having a secure attachment but still have the symptoms of disinhibition that are related to the disrespect of social boundaries. Scholars in the field argue that the symptoms of disinhibited attachment represent a failure of the previously institutionalised child to form a selective, particular attachment relationship. Since this pattern only exists when institutional deprivation persists beyond six months of age, there is likely a sensitive period for attachment relationship formation [8].

There is evidence that attachment disorganisation is associated with various disturbances in childhood [13,14,15,16,17,18,19]. It is also known that interventions are needed to help move from disorganised to secure attachment [20]. Research shows that early interventions (up to the age of 5) with primary caregivers have a positive impact on children’s disorganisation [21]. However, the research evidence on interventions for institutionalised children is limited and heterogeneous. There are few studies reporting positive effects of individual therapies: equine therapy improved children’s self-regulation and self-control in [22], and art therapy improved self-esteem and reduced emotional and behavioural problems in [23]. In the search for interventions that focus on improving the attachment security of institutionalised children, the evidence is even more scarce. One long-term intervention (from 4 to 9 or more months of exposure) delivered by regular institutional staff was conducted with young children aged ~6 months to 4 years [24]. The results of this research supported the theory that a responsive, consistent relationship with previously trained regular caregivers has a positive impact on children’s development and attachment [24,25,26]. There may be a lack of studies for institutionalised children because it is strongly recommended that children spend as little time as possible in institutions [3]; therefore, the most meaningful intervention for them is placement in a family environment through reunification with their biological family, adoption, or fostering. There is continuing evidence of the positive effects of adoption after institutionalisation. Very often, especially for children adopted early, later life outcomes do not differ from those of children cared for in biological families [11,27,28]. Positive outcomes have also been found for previously institutionalised children placed in foster care [29,30,31,32]. However, despite the ongoing deinstitutionalisation process in Europe and worldwide, some children continue to grow up in institutions for various reasons. As mentioned above, these children often face different emotional, behavioural, and attachment challenges and, therefore, require interventions. One such intervention will be described in this paper.

### 1.2. Current Study

The main aim of this study is to describe the changes that took place after a one-year psychosocial intervention in a daycare centre for children from institutions with various behavioural and emotional disorders.

Our main hypotheses were as follows:

1. Positive changes in children’s mental health will be observed after the intervention.
2. On the CAI, the subscales indicating security of attachment will shift towards being more secure.

## 2. Materials and Methods

### 2.1. Study Design

A case-series study design was employed, as it was deemed to be the most suitable for a purposive sample. The present study employed a descriptive case analysis of the data collected from the five participants prior to their entry into the psychosocial rehabilitation programme and following one year of participation. The two cases in which the organisation of attachment underwent a change after one year will be described in greater detail.

### 2.2. Participants

The children were referred to the programme by their child psychiatrist from the outpatient mental health centre. The following criteria were established for eligibility in the study:

1. Participation in the programme during the study year.
2. Children aged 8–11.
3. Children lived in institutional care during their participation in the programme
4. Children may be diagnosed with an emotional, behavioural, attention deficit, or learning disorder but not an intellectual disability, autism spectrum disorder, or psychosis.
5. Legally responsible persons accept the participation of the children.
6. Children consent to participate in the study.

Consequently, seven participants (two female and five male) were included in the study, for whom the legally responsible persons signed informed consent forms. The main researcher explained participation in the study to each child individually, and the children also signed a short consent form written in simple language. In accordance with the ethical standards and to ensure the confidentiality of the data, each participant was assigned an identification number at the beginning of the study, which was only known to the principal researcher. Two boys dropped out; they refused to be filmed during the CAI. 

### 2.3. Sampling and Setting

The study was conducted at a recently inaugurated psychosocial rehabilitation centre, established by the municipality in Vilnius, Lithuania, which represents a distinctive and pioneering initiative in the country. The centre is located in Vilnius, specifically in a quiet, wooded area, which means it is situated at a distance from the city centre. It is a separate individual building fully equipped to meet both the physical and emotional needs of the children, with sensory, calming rooms, as well as dedicated spaces for art and music therapies. The programme can serve up to six clients at a time. This study, therefore, had a purposive sample. In the preceding section, the eligibility criteria for participants were defined. The centre was initially set up to help children growing up in institutions. The study was carried out between 2019 and 2021. The participants were included consecutively. 

The same researchers conducted another similar case series study in a selected social daycare centre, but the participants lived with their own families. The results of this study have been published elsewhere [33]. 

### 2.4. Assessment

Assessments were scheduled at baseline (T1) and after one year of attending the centre (T2). The same procedure of assessment was used at T1 and at T2. We used the questionnaires that are translated and standardised in Lithuania. In order to assess attachment, we chose the Child Attachment Interview method, which will be described further.

1. Standardised Lithuanian translations of the ASEBA (Achenbach System of Empirically Based Assessment) questionnaire and the Child Behavior Checklist for Parents (CBCL/6–18) and Teachers (TRF/6–18) were used [34].
2. Four–seventeen full and follow-up versions of the Strengths and Difficulties Questionnaire (Lithuanian translation) were used, along with the impact supplement for caregivers and teachers (SDQ-Lit).
3. A semi-structured clinical psychiatric interview for caregivers was used. This interview was developed by the first author of this paper, M.M., a child psychiatrist, according to the guidelines indicated in JM Rey‘s IACAPAP e-Textbook of Child and Adolescent Mental Health [35]. The interview consisted of 17 open questions, with clarifying subquestions if needed. M.M. conducted all the interviews with the caregivers—the person closest to the child from the institution.
4. The Child Attachment Interview (CAI) method was used for children at T1 and T2, and a Lithuanian interview translation was used [36]. The CAI was developed by Shmueli-Goetz et al. from the Anna Freud National Centre for Children and Families as a measure designed to assess attachment in middle childhood and adolescence [37,38,39]. In order to adhere to the requirements of CAIs, the children were interviewed by the principal researcher at T1 (when the children did not know her) and by the research collaborators, who had received 3 h of training on how to conduct CAI. The interviews were video-recorded, as coding needs to be based not only on spoken language but also on non-verbal behaviour [37,40]. After the transcription of the CAI, it can be coded by a certified coder, where a score is given according to the following nine scales: Emotional Openness, Balance of positive and negative references to attachment figures, Use of examples, Preoccupied Anger, Resolution of Conflicts, Idealization, Dismissal, Atypical/Disorganized Behavior, and Overall Coherence. Emotional Openness, Balance of positive and negative references (to attachment figures), Use of examples, Resolution of Conflicts, and Overall Coherence scales scores of 5 and above to 9 indicate attachment security, and scores below 5 indicate insecurity. Finally, attachment organisation was determined; a child can be classified as secure, insecure dismissing, insecure preoccupied, and disorganised. A different attachment classification was designed for all attachment figures.
5. We used clinical mental status examination interviews for the children, designed with the expertise of the principal researcher. M.M. structured these interviews according to the guidelines indicated in JM Rey’s IACAPAP e-Textbook of Child and Adolescent Mental Health [35]. Each interview consists of 22 questions, with subquestions if needed.

### 2.5. Data Collection Time and Data Processing

Data collection started in February 2019 and ended in August 2021, which included the COVID-19 pandemic and quarantine period in the country. The first 4 participants, who joined the study in February 2019, attended the centre without interruption until February 2020. The last participant joined the study in September 2020. Due to quarantine requirements, he experienced more individual virtual time than live group work.

M.M. analysed all the data from the CBCL6/18, TRF6/18, and SDQ questionnaires and the interviews with caregivers and children. Descriptive data analysis was performed based on the questionnaires, CAIs, and clinical interviews. The CAIs were transcribed by M.M. and her collaborators and coded by an independent certified CAI coder. The second certified CAI coder coded thirty per cent of the interviews to test the reliability of the coding, and the two coders were blind to each other’s scores. There was 100 per cent agreement between the two coders on the attachment classification. Intraclass correlation coefficients (ICCs) between the two coders were substantial for eleven subscales, ranging from the lowest ICC for Resolution of Conflicts of 0.64 to values of 0.7 to 0.92 for the other subscales. The median ICC indicated robust agreement between the two coders; for all scales, it was 0.86.

### 2.6. The Content of the One-Year-Long Psychosocial Intervention in the Daycare Centre

The intervention consisted of individualised care focusing on staff building a trusting relationship with participants using the principles of Trust-Based Relational Intervention^®^ (TBRI). The children attended the daycare centre four to five days a week after school for 3 h a day. The therapeutic programme for each participant was individualised according to each child’s difficulties but was similar for all. The ratio of children to staff was ~0.7 to 1. All children had a staff member who was assigned to be that child’s “own person”. The “own persons” were social workers. They established a secure and trusting relationship with their assigned child and were responsible for coordinating and individualising the therapeutic programme. Individualisation involved the “own person” and the child working together to establish individual goals for the intervention. These goals could be as diverse as the creation of a book detailing the child’s life story, and they were continually adapted in accordance with the individual progress of each child. Each “own person” had two children in their care. The participating children also had relationships with other members of staff, including psychologists, art and music therapists, and social workers. Staff communication and therapeutic relationships were based on the TBRI principles. All staff received 22 h of TBRI training from the lead researcher, a certified TBRI practitioner. They then attended TBRI case study sessions twice a month for 3 h each for a year. During the case study sessions, the responses to each child’s needs were discussed on an individual basis. TBRI is a holistic, attachment-based, trauma-informed, evidence-based intervention grounded in neurodevelopmental knowledge and designed to meet the complex needs of vulnerable children [41,42,43]. The fundamental principle of TBRI is that the rebuilding of trust through the establishment of a caring and skilful relationship with an adult can facilitate the healing of children from adverse experiences [41]. A summary of the principles of TBRI is provided in Table 1. For further details regarding the TBRI training programme, please refer to the following link: https://child.tcu.edu/professionals/tbri-training/#sthash.udYEr7GT.dpbs (accessed on 10 July 2024).

In addition, each child underwent a weekly one-to-one session of either art or music therapy, conducted by the relevant specialist. They also had individual psychotherapy sessions once a week in the centre or continued sessions with their previous psychotherapist outside the centre. Furthermore, the children engaged in collective activities with staff members (e.g., reflection sessions, sensory and physical activities, and at mealtimes). Those group activities were also influenced by the TBRI principles, with the objective of identifying the needs of the child beyond their behavioural manifestations and responding to those needs. All of the children’s main caregivers (most often, they were the principal staff members from their institution) had a meeting with the child’s “own person” and psychologist every six weeks. They discussed the child’s emotions, behaviour, and communication habits. The carers gained insight into the child’s behaviour and what helped them to deal with their particular problems. The meetings were informative, supportive, and aimed at providing practical recommendations for the carers. The carers did not receive TBRI training, except for those of the child who joined the study in September 2020. This child’s two main caregivers received 22 h of TBRI training.

It should be noted that the precise details regarding the therapeutic hours for each participant can be made available upon request from the corresponding author to any person with an interest in this matter.

## 3. Results

### 3.1. Descriptive Data

The participants in the study were five children aged 8–11 years (mean = 9.4, SD = 0.9). All of the participants had a history of adverse childhood experiences, and several of them had actual negative relationship experiences, as reported by their caregivers (see Table 2). 

**Figure 1:**
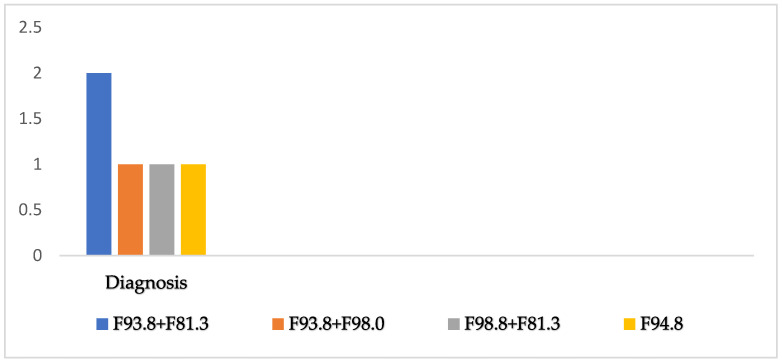
Participant diagnoses, according to ICD-10.

All participants had a psychiatric ICD-10 [44] diagnoses prior to entering the centre (see Figure 1). Three were diagnosed with other childhood emotional disorder (F93.8); one with other childhood social functioning disorder (F94.8); and one with attention deficit disorder without hyperactivity -F98.8. Three children had a second diagnosis of mixed disorder of school skills (F81.3), and one boy had a second diagnosis of non-organic enuresis (F98.0). The diagnostic categories show the difficulties these children had before entering the psychosocial rehabilitation centre. According to the data from the clinical interviews with the caregivers, the children had episodes of aggression; the children were anxious and/or stressed; some of the children had self-harming behaviour and also difficulties in communicating with peers; and some of the children had difficulties in concentrating, sitting still during lessons, and a lack of motivation to learn.

The distribution of participants’ attachment classification at T1 was as follows: three children were classified as insecure dismissing, and two were classified as disorganised. The classification was the same for all attachment figures; most often, children reported biological parents or grandparents as their attachment figures. 

The data on difficulties at school were as follows: Four participants had already been assessed by the Pedagogical Psychological Service (PPS) and diagnosed with a learning disability, including attention problems, speech and language problems, low intellectual functioning, and/or specific reading and writing problems. One participant was still waiting to be assessed by the PPS. The questionnaires completed by the teachers showed a slightly different picture: in TRF6/18, only one participant had a clinical level of aggressive and rule-breaking behaviour. According to the data of the SDQ-, two participants had attention difficulties at school. However, during the interviews, all carers reported that the children had either learning difficulties (attention problems, unwillingness to learn, distractibility) or behavioural difficulties at school (conflicts with peers, oppositional and challenging behaviour towards teachers).

### 3.2. The Change after One Year

Data from the clinical interviews with caregivers at T2 showed that all five participants improved in behaviour, emotional and academic functioning, and communication. The carers reported that the children were less anxious, had fewer outbursts of anger, were more able to regulate their emotions, and were able to verbalise their feelings. The carers also reported that communication with the children became easier, that there were fewer conflicts with the children, and that the children expressed less disruptive behaviour. In terms of improved academic functioning, the caregivers reported that the children had better concentration, more motivation for learning, quicker homework completion times, and fewer conflicts with teachers and other children at school.

One participant’s attachment organisation changed from disorganised to insecure dismissing, but for one participant, his insecure dismissing attachment shifted to disorganised at T2. Two participants showed a slight improvement on the CAI scales, indicating security. This improvement in the security scales was very small and did not reach a score of 5, which would indicate more consistent security. The changes in the security-indicating scales between T1 and T2 after the CAI are shown in Table 3. 

At T2, lower scores were observed on most scales of the CBCL6/18 questionnaire compared to T1 (see Table 4), but according to the teachers, higher scores were observed at T2. This shows that the caregivers from the institution marked that the children had improved: they had less anxiety, fewer social problems, fewer problems with thinking and attention, and less aggressive and rule-breaking behaviour. The teachers reported more attention problems and more symptoms of withdrawal at T2. This difference can be explained by the fact that different teachers completed the questionnaires for several participants.

At T2, lower scores were also observed on most scales of the caregiver and teacher forms of the SDQ (see Table 5). We can see that the carers reported fewer symptoms of emotional distress, fewer behavioural difficulties, and fewer difficulties with peers. Teachers, on the other hand, reported less hyperactivity and concentration difficulties and fewer peer difficulties, but they saw slightly more emotional distress and behavioural difficulties.

A shift in attachment organisation was noted for two participants; these cases will be discussed in more detail. 

### 3.3. The Case of Adam (Pseudonym)—9-Year-Old Boy

At T1, Adam suffered from chronic symptoms of severe outbursts of anger, with physical aggression towards things and people; sexualised behaviour; negative attitudes towards himself; an irritable mood; problems with peer communication; learning problems; and nocturnal enuresis, as reported by his caregivers. These symptoms were reported in the questionnaires: the caregivers reported problems on all scales of the SDQ, and on the CBCL6/18, and the Anxiety/Depression, Social Problems, Thinking Problems, Rule-Breaking Behaviour, and Aggressive Behaviour scales were in the clinical range. The teacher noted behavioural difficulties and difficulties getting along with peers on the SDQ, and problems communicating with peers and a lack of motivation were noted on the TRF6/18. The boy himself reported having enuresis. 

Adam had lived in residential care since the age of five and a half. He had a history of physical and emotional neglect and probable sexual abuse. His mother was addicted to alcohol and his father to opioids. 

His classification of disorganised attachment at T1 changed to insecure dismissing at T2. This change is consistent with his clinical improvement—his carers reported a remarkable change in his behaviour. He also had much fewer episodes of enuresis. On the CBCL6/18 at T2, the caregivers reported only Thought Problems in the clinical range and Aggressive Behaviour in the borderline range. The teacher reported borderline Rule-Breaking Behaviour on the TRF6/18. 

This boy participated in the therapeutic programme for 55 days face to face and 66 days virtually because of the COVID-19 pandemic. He had supportive factors that could contribute to his marked improvement. During the study, he met his future foster parents. His fostering was completed with the conclusion of the legal process of preparing him to move in with them. In the interview, he spoke of them as his future parents, and his residential carers reported good and stable relationships with them. This boy continued (for the third year) to have weekly sessions with his previously known psychotherapist. His two primary caregivers from his institution had completed TBRI training and were trying to apply the intervention principles in the institution, so this boy experienced consistency in two settings: the psychosocial rehabilitation centre and the institution.

### 3.4. The Case of Thomas (Pseudonym)—10-Year-Old Boy

At T1, Thomas had chronic and severe outbursts of anger, with physical aggression towards objects and people; a depressed mood, self-harming behaviour; difficulties adapting to the environment; and learning problems. Using the SDQ, the carers reported behavioural difficulties and difficulties getting along with peers at moderate risk. The teacher, on the TRF6/18 form, noted that the boy was impulsive, lacking in self-confidence and motivation, and had attention problems. 

Thomas was removed from his biological family at the age of 1. He had experienced physical and emotional neglect during the first year of his life. His mother had a history of alcohol abuse. He had witnessed conflicts in the family. His biological parents maintained a rare and fractured relationship with him until the boy was ten, when they had legal proceedings for the restoration of parental rights. At the age of nine, before entering the psychosocial rehabilitation centre, Thomas experienced relational trauma—he had changed institutional home and changed staff. 

He was classified as insecure dismissing at T1 and moved to disorganised attachment at T2. His scores on the security subscales were also lower at T2, with the exception of the Resolution of Conflicts subscale. This boy had an exceptional experience in residential care—his parents asked for permission to take him home after 9 years. This act of the parents coincided with the therapeutic process in the psychosocial rehabilitation centre. Thomas’s assessment at T2 took place only one month before he moved in with his biological parents. 

His carer from the institution reported an improvement at T2: he had fewer outbursts of anger; he could accept compromises; and he was more engaged in learning. This boy also said in the interview that he could now regulate himself better. During the study, this boy changed schools—he finished primary school and moved to secondary school. At T2, the new teachers had reported that Withdrawn/Depressed was in the clinical range and Aggressive Behaviour and Anxiety/Depression were in the borderline range. Thomas participated in the face-to-face therapeutic programme for 107 days.

## 4. Discussion

We aimed to describe the possible change in the psychosocial intervention applied in the daycare centre for one year, focusing on whether it could influence the attachment security and general mental health of children from institutional care. We expected that the symptoms of mental disorders would improve and that the scales indicating security in the CAI would shift towards being more secure. Bearing in mind the limitations of our study (chiefly, the very small sample), the results suggest that behaviour, emotional functioning, learning skills, and communication with others improved for all five participants. One participant’s attachment organisation changed from disorganised to insecure dismissing. However, another boy’s insecure dismissing attachment shifted to disorganised, although he had also improved clinically. We will discuss our findings below.

Efforts to increase participants’ attachment security were the main focus of the psychosocial intervention, so the ratio of staff to children was 0.7 to 1. One staff member was assigned to form a therapeutic alliance with each child. We know from research that even when children live in institutions, outcomes are better when the staff–child ratio is relatively high, as it is expected that the staff will be more sensitive towards their relationships, and this has been demonstrated in a longitudinal study [24,25,26]. We have observed that most of our participants lacked a stable and responsive relationship, so participation in the programme could address this need.

As we noted in the introduction, there is very little research on the effects of psychosocial interventions on the attachment of institutionalised children. However, we know from research with adults that therapeutic relationships and emotional support from others can enhance attachment security [45,46]. Our study participants had this stable and responsive relationship while participating in a programme; therefore, this relationship may have contributed to the improvement of their mental health and attachment security

We were not able to assess the attachment style of the staff. However, the programme coordinator was attentive to the practices that could help staff to be mindful in their interactions. Staff had reflection time and individual and group supervision, so they could explore their feelings and relationship history. We know from research in foster care that when children are placed with foster parents who have an autonomous attachment style, they can form organised attachment relationships [47]. Therefore, in the therapeutic programme, there were practices mentioned earlier that could help staff to become more autonomous. There is research that shows that when staff members build trusting relationships, it can be similar to responsive parenting; therefore, we can expect improvements in attachment security and mental health [26]. In our study, the TBRI training taught staff how to build a trusting relationship and respond to a specific child’s needs, which could be compared to the principles of responsive parenting. 

The authors of this paper conducted a further case series study on the impact of a targeted psychosocial intervention (TBRI) on traumatised children aged 8–11 years. Preliminary evidence suggests that in middle childhood, with the help of TBRI for several children, disorganised attachment turned into insecure dismissing, though these children were living with their biological families [33]. To our knowledge, there are no published data on interventions aimed at improving attachment in this age group for institutionalised children. Although the results of our study should be treated with caution due to the limitations of the study, the study observations suggest that improvement in attachment security can be achieved in this age group also.

From the descriptive part of the study results, we found that four participants had lived in the harsh environment of their biological families for four to six years, and all of them had lived in institutional care for more than four years. We know from research that if the harsh environment lasts as long as in these cases, we can expect these children to develop a range of emotional and behavioural problems [2,3,5,6,7]. Our participants all had clinically significant symptoms of emotional and behavioural disturbances. There was no secure attachment classification; therefore, two participants were classified as disorganised at T1. 

For one participant (pseudonym Adam), we saw a remarkable clinical improvement, and his attachment moved from disorganised to insecure dismissing. However, he had additional supporting factors that may have contributed to this marked improvement. Prior to attending the daycare centre, this boy had been in individual psychotherapy for two years, although symptoms of emotional disturbance were still present. We know from research that traumatised children, as they develop disturbances in different areas of functioning, require a complex intervention that responds to their specific needs [41,48,49]. Therefore, it may be the case that one hour of psychotherapy is not enough for such highly disturbed children, as we witnessed in this boy’s case. During the study year, this boy met his future foster parents, and we felt that this hope of moving to live with them might also contribute to this child’s healing. A qualitative study would be needed to test this hypothesis. Two of the carers from this boy’s home attended the TBRI training during the study. It is therefore possible that their relationship with the child also helped him to improve.

Despite the clinical improvement, we have seen that the second boy (pseudonym Thomas) has been classified as disorganised at T3. Thomas’s experience was exceptional in the institutional care system. He was preparing to be reunited with his biological parents after nine years in the institution. His mind showed signs of disorganisation even though he was preparing to move home as Adam. We could speculate that this event was both joyful and stressful, prompting memories of negative experiences in early childhood. Research on children in foster care shows that reuniting with biological parents does not solve problems per se [50,51]. Thomas’s biological parents received counselling during his participation in the programme to prepare Thomas for the transition, although we do not have data on Thomas’s mental health after placement.

For three other participants, we saw a slight clinical improvement. For two of them, a slight improvement in the security subscales pertaining to the CAI was also noticed. Those three children endured relational trauma during the study; they participated in a foreign foster families project and went to a foreign country for the holidays with a foster family. They experienced rejection by temporary foster families and have been placed into new ones. This experience negatively impacted our study participants, as reported by their caregivers. Research in foster care shows that ruptured placements negatively affect children’s emotions and behaviour [52]. It is known that healing cannot occur without a stable, consistent, nurturing relationship with a sensitive and responsive adult [42,52]. There exist data from qualitative research with Latvian institutional care leavers who reported that the opportunity to have a caring relationship with staff was an important factor for them [53]. Research shows that the caregiver’s commitment also plays an important role in the child’s sense of security [47]. Therefore, the practical implications of our findings suggest that any new relationship with an adult should be entered into only after an assessment of whether the relationship can be sustained has been made. Adults entering into a relationship with a child from an institution should demonstrate an autonomous attachment state, as relating to a child with a traumatic history requires specific knowledge and resources, as has been demonstrated with adoptive parents [54]. 

Upon the CAI, it was expected to observe an improvement in the subscales indicating security. In the majority of participants, a slight improvement was noted in the security subscales. An important finding is that we saw a shift from disorganised to organised classification in one child. Although the findings are only those of an observational study with inherent limitations, they are consistent with those of other researchers who have argued that the attachment system is responsive to the experience of relationships [55]. We also know that children who have had difficult experiences in the past need at least one stable and supportive relationship to be resilient [56]. From our participant group, this was very true for Adam; he had several stable, supportive, and enduring relationships.

## 5. Limitations

A major limitation of this paper is the small sample size. The case series design also has limitations; we did not have a control group, so we can only observe the tendency and preliminary evidence indicating that a change occurred after participation in the psychosocial rehabilitation programme. The use of a comparison group could shed light on the findings regarding possible developmental and natural changes in attachment and mental health. Future research using more rigorous designs will be needed to investigate how psychosocial interventions might affect attachment and mental health.

## 6. Conclusions

Our study aimed to describe the possible impact of a psychosocial intervention applied in a daycare centre on the attachment security and mental health of institutionalised children. Taking into account the limitations of the study design, we observed an improvement in the mental health of all children. For one boy, his disorganised attachment changed to insecure dismissing, and for the majority of children, the security subscales improved slightly. Two participants had life-changing events during the study—they were preparing to move into families. The other three suffered broken relationships with their temporary foster carers. Despite these complexities, we saw improvements in participants’ mental health and attachment security. However, as the sample size is small, we are not able to link the improvement to the intervention alone. However, in the psychosocial rehabilitation programme, the children received nurturing and individualised care, which allowed staff to analyse each case thoroughly. Participation in the programme is likely to have helped the children to cope more smoothly with their life-changing events. The results speak to the complexity of each child’s situation and the fundamental need for an adult who can provide in-depth care. Staff in institutions are often unable to meet this need, so the programme staff were able to fulfil this role even though they only saw the child a few times a week. As children in care have a history of adverse life events and repeated relationship breakdowns can be damaging, the study findings suggest that any new and intensive relationship with an adult for them should be established after an assessment of whether the relationship has the potential to be sustained. Bearing in mind the limitations, the study findings suggest that psychosocial interventions based on building individual trusting relationships with the child may help improve the mental health and attachment security of institutionalised children.

## Figures and Tables

**Table 1:** TBRI principles [33].

Connecting Principle	Empowering Principle	Correcting Principle
Engagement Strategies—teaching to connect with children, such as with eye contact, behavioural matching, and playful engagement. Mindfulness Strategies—training caregivers’ awareness of what do they bring to the interactions with children and teaching the importance of self-care.	Physiological Strategies which focus on the internal physical needs of the child (hydration, nutrition) and sensory needs. Ecological Strategies that focus on the child’s external environment (transitions, scaffolding, daily rituals) and learning self-regulation skills.	Proactive Strategies—designed to teach social skills to children during calm times. Responsive Strategies provide caregivers with tools for responding to challenging behaviour from children.

**Table 2:** Participants’ adverse childhood experiences and negative life events.

Participant ID	Adverse Childhood Experiences and Negative Life Events
01	► Physical and emotional neglect in the biological family ► Witnessed physical violence in biological family ► Removed from biological family at age 4 ► Relationship trauma—had 2 different temporary foster families
02	►Emotional and sexual abuse ►Removed from biological family at age 6 ►Repeated relationship trauma—had 4 different temporary foster families
03	► Physical and emotional neglect in the biological family ► Witnessed physical violence in biological family ► Removed from biological family at age 5 ► Repeated relationship trauma—had 2 different temporary foster families
06	► Physical and emotional neglect in the biological family ► Witnessed parental conflict in biological family ► Removed from biological family at age 1 ► Relationship traumatised aged 9—placed in another home
019	► Physical and emotional neglect within the biological family ► Emotional and sexual abuse in the biological family ► Removed from biological family at age 5.5 ► Bullying at school

**Table 3:** Scores on scales indicating security at T1 and T2.

Participant ID	EO_T1	EO_T2	Bal_T1	Bal_T2	Ex_T1	Ex_T2	Confl_T1	Confl_T2	Coh_T1	Coh_T2
01	1.5	1.5	3	3	2	1	5	3	2	2
02	2	2	2	4	2	4	2	3	1	3
03	1	1.5	1	2	3	3	3	1	2	2.5
06 *	2.5	1.5	2	1	3.5	2.5	3	4	3.5	1
019 **	3.5	2	4	1	3	2	3	3.5	3	3

EO stands for Emotional Openness, Bal—Balance of positive and negative references to attachment figures, Ex—Use of examples, Confl—Resolution of Conflicts, Coh—Overall Coherence. * attachment classification changed from insecure dismissing at T1 to disorganised at T2. ** attachment classification changed from disorganised at T1 to insecure dismissing at T2.

**Table 4:** General trends in symptoms as measured by the CBCL6/18 and TRF 6/18 at T1 and T2.

Subscale	T1 M (SD)		T2 M (SD)	
	CBCL	TRF	CBCL	TRF
Anxiety/Depression	7.4 (6.88)	3.2 (2.39)	4.0 (2.54)	3.6 (4.77)
Withdrawn/Depressed	3.0 (1.58)	2.2 (0.84)	3.4 (1.67)	3.8 (4.65)
Social Problems	6.6 (3.84)	3.0 (1.22)	5.0 (2.12)	2.6 (1.51)
Thought Problems	3.2 2.39	1.2 (0.45)	1.0 (0.0)	1.4 (0.89)
Attention Problems	6.8 (1.64)	16.6 (4.51)	6.2 (1.31)	19.0 (4.00)
Rule-Breaking Behaviour	8.8 (4.97)	4.2 (2.17)	5.0 (1.87)	4.2 (3.7)
Aggressive Behaviour	14.2 (9.76)	5.2 (2.59)	10.6 (3.78)	8.6 (9.07)

**Table 5:** General trends in symptoms as measured by the SDQ from T1 to T2.

Subscale	T1 M (SD)		T2 M (SD)	
	Caregivers	Teachers	Caregivers	Teachers
Emotional distress	3.2 (2.28)	1.4 (0.54)	2.4 (2.19)	1.6 (1.34)
Behavioural difficulties	3.8 (2.38)	2.2 (0.44)	2.8 (0.83)	3.0 (1.58)
Hyperactivity and concentration difficulties	6.4 (2.3)	5.8 (2.58)	6.4 (2.19)	4.8 (1.78)
Difficulties getting along with other children	4.4 (0.54)	2.6 (1.14)	3.8 (1.3)	2.2 (0.83)

## Data Availability

The data that support the findings of this study are available from the corresponding author, M.M., upon reasonable request.
